# Socioeconomic and sociodemographic factors associated with food expense insufficiency during the COVID-19 pandemic in Japan

**DOI:** 10.1371/journal.pone.0279266

**Published:** 2022-12-15

**Authors:** Ryoko Katagiri, Takahiro Tabuchi, Kota Katanoda

**Affiliations:** 1 Division of Epidemiology, Institute for Cancer Control, National Cancer Center, Tokyo, Japan; 2 Cancer Control Center, Osaka International Cancer Institute, Osaka, Japan; 3 Division of Surveillance and Policy Evaluation, Institute for Cancer Control, National Cancer Center, Tokyo, Japan; Sri Lanka Institute of Information Technology, SRI LANKA

## Abstract

**Objective:**

To examine the status of food expense insufficiency in Japan during the coronavirus disease 2019 (COVID-19) pandemic and the socioeconomic and sociodemographic factors associated with food expense insufficiency.

**Design:**

Food expense insufficiency before and after the pandemic was assessed. The multivariable-adjusted odds ratio (AOR) and 95% confidence interval (CI) were calculated for the association between food expense insufficiency and socioeconomic and sociodemographic factors.

**Setting:**

A large-scale, cross-sectional online questionnaire survey.

**Participants:**

From August to September 2020, 25,482 participants aged 15–79 years completed the questionnaire (JACSIS 2020 study; Group 1). In October 2020, 917 single parents were surveyed for oversampling purposes. There were 179 single parents in Group 1 and a total of 1096 single parents in Group 2.

**Results:**

Group 1 and Group 2 had 747 (2.9%) and 55 (5.0%) participants, respectively, who experienced food expense insufficiency for the first time after April 2020. Young age, part-time employment, being a single parent (in Group 1), and the number of people in the household (five or more in Group 1 and child/children alone in Group 2) were significantly associated with food expense insufficiency. As being a single parent was significantly associated with food expense insufficiency in Group 1 (AOR [95% CI] = 7.23 [5.40–9.68]), we further examined it in Group 2. Single parents who exhibited multiple factors (young age, part-time employment, living only with child/children) were likely to experience food expense insufficiency (15.3–15.8%).

**Conclusions:**

Triggered by the pandemic, a small percentage of individuals experienced food expense insufficiency. We identified that factors such as young age, part-time employment, and being a single parent were significantly associated with food expense insufficiency, and discovered that a multiplicity of these factors further increased the risk. Our findings suggest an urgent need to support individuals with a potentially high risk of food expense insufficiency.

## Introduction

The coronavirus disease 2019 (COVID-19) pandemic continues in the middle of July 2021; according to the World Health Organization COVID-19 dashboard, over 195,000,000 cases have been confirmed globally [1]. Due to COVID-19, health inequality has been the focus of attention because of the disproportionately high mortality in ethnic minority groups [2]. Moreover, although a new working style, such as working from home, becomes an option to continue working during movement restrictions, this option is not equally available for all employees and has the potential to exacerbate inequalities [3, 4]. In addition to the high unemployment rate due to the COVID-19 pandemic [5, 6], these situations affect household income [4] and lifestyle, including access to food.

Food insecurity is defined as a situation in which access to desirable food is threatened and is often focused on in the context of global health [7]. Food expense insufficiency is one cause of food insecurity. According to a review of high-income countries, food insecurity measures focus on the access dimension of food insecurity because constraints mainly exist in the access dimension in high-income countries [8]. Food access is limited by both physical and economic constraints. As a lack of expenses might be a constraint for food access under the COVID-19 pandemic, we focused on food expense insufficiency in this survey. Overall, food insecurity has received global attention during the COVID-19 pandemic. While several papers have identified vulnerable groups in terms of race or economic status in countries such as the US, Australia, and Nepal, the situation in some other countries remains poorly documented [9–12]. Although studies on the association between socioeconomic status and dietary intake or health outcomes have been published in Japan [13, 14], the prevalence of food insecurity, food expense insufficiency, and related factors are still unknown. Further research focusing on food insecurity, including food expense insufficiency, is required to develop food-based welfare policies or other safety nets to maintain a suitable population nutrient status.

According to the World Bank, Japan has one of the lowest poverty rates in the world (0% in 2010, 0.7% in 2013) [15]. Despite these low poverty rates in the global survey, the relative poverty rate (household equivalent income ≤ 1,270,000 yen/year [approximately £ 8,500]) was 15.4% in a domestic survey in 2018 [16]. During the COVID-19 pandemic, economic access to food from these households might have been affected. Therefore, the study results on food expense insufficiency and relevant factors from countries with low poverty rates, such as Japan, may add some evidence to address food access during the COVID-19 pandemic. This could be a reference for other high-income countries.

Using a large cross-sectional internet survey, we examined the prevalence of food expense insufficiency, a dimension of food insecurity [17], and investigated the sociodemographic status of participants with food expense insufficiency triggered by COVID-19. This study aimed to clarify the characteristics of vulnerable populations facing food expense insufficiency.

## Method

### Study design

**Fig 1:**
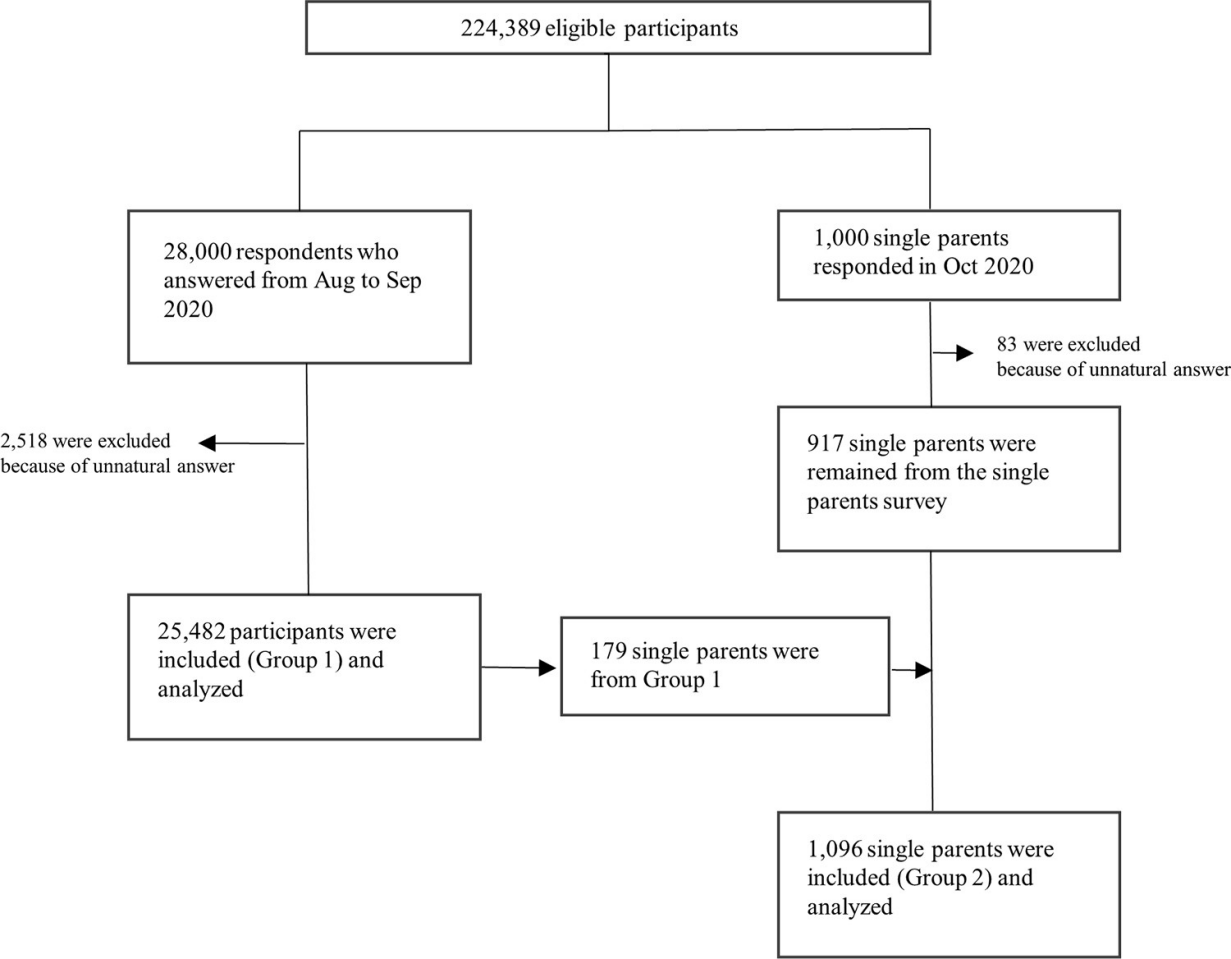
Flowchart of participant selection.

The Japan COVID-19 and Society Internet Survey (JACSIS) is a web-based, cross-sectional survey using a self-reported questionnaire. This type of internet survey has been used in previous studies, and the details have been described elsewhere [18, 19]. In this analysis, two types of JACSIS surveys, a survey of people living in Japan (Group 1) and a survey of single parents, were included (Fig 1).

The survey procedure for Group 1 was as follows: First, the survey requests were sent to potential participants aged 15 to 79 years by a large Internet survey agency (*Rakuten Insight*, Inc., where approximately 2.2 million people were registered in 2019). The number of eligible participants in this study was 224,389, and they were selected randomly according to sex, age, and area (prefecture). Second, the selected participants provided consent to answer the questionnaire and did so on the website. They were able to stop answering or skipping questions at any point during the survey period. The survey of Group 1 was completed between August 25 and September 30, 2020; by the latter date, the number of participants who answered the questionnaire reached the predetermined numbers in each category of sex, age, and area (28,000 respondents were defined as a sufficient number at each age, sex and area stratum to estimate the proportion of events and available budget for the survey; response rate 12.5%). To maintain data quality, participants whose answers showed discrepancies or were unrealistic were excluded (N = 2,518). Discrepancies were detected for the following three questions: “Please choose the second box from the bottom” (participants who selected the wrong answer were excluded), “Please choose all recreational substances and medications (e.g., legal/illegal opioids, cocaine, cannabis, anxiolytic agents and medication for sleep) you use” (participants who indicated that they were using all were excluded), and “Please choose chronic diseases (e.g., cancer, diabetes, stroke, ischemic heart disease, asthma, mental disease) you have” (participants who selected all diseases were excluded). Some participants under 20 years of age may be vulnerable to changes in social conditions due to the COVID-19 pandemic; therefore, we did not exclude them. In total, 25,482 participants were included in the survey group.

As single parents may be susceptible to changes associated with COVID-19, such as economic trends or employment status, we surveyed them for oversampling. The single-parent survey was conducted from October 16, 2020, to the time when the number of participants reached 1,000 (October 23, 2020). Single parents in this survey were defined as “Participants who are living with two or more family members (including themselves),” “have no spouse,” and “have child/children aged 0–14 years old.” The same quality control procedure was followed for these data as in Group 1, resulting in a final sample of 917 participants. Using the same criteria for single parents, we selected single parents from Group 1 (n = 179) and combined them with the 917 participants; in total, 1096 single parents were defined as Group 2 in this analysis (Fig 1).

### Ethics

This study was conducted in accordance with the guidelines of the Declaration of Helsinki, and all procedures involving research study participants were approved by the Ethics Committee of the National Cancer Center Japan (2020–447) and the Osaka International Cancer Institute (no. 20084). Web-based written informed consent was obtained from all patients, including those aged < 20 years. According to the Japanese ethical guidelines, a study participant who has completed middle school and above is subject to their consent. Although this study included participants under 20 years of age, all participants had completed junior high school and were aged 15 years or older at the time of the survey. The inclusion of participants aged 15 years was judged by the ethical committee, and we obtained approval from the Research Ethics Committee of the Osaka International Cancer Institute and National Cancer Center Japan, including the informed consent process for these minors. Moreover, since this study was not invasive, we obtained the approval of these two institutional review boards, information including the purpose of the research was made public and opportunities to refuse the research were ensured.

### Variables

The surveys for both Groups 1 and 2 used the same questionnaire. For food expense insufficiency, we asked, “Have you experienced an episode of food expense insufficiency since April 2020?” and the participants were instructed to select one of three choices: “1. Experienced for the first time,” “2. Experienced before April 2020 and continued,” or “3. Not experienced.” In April 2020, the first “state of emergency” for COVID-19 was declared and all schools in Japan were closed. The responses to these questions before and after April 2020 captured the impact of this situation. The participants were asked about their age, sex, and area (prefecture). The area classification was according to the number of COVID-19 patients until April 16, 2020: “Over 10 patients/100,000 population area,” “5–9.9 patients/100,000 population area,” or “less than 5 patients/100,000 population area.” Participants were further categorized by achieved education level (“high school or lower” and “college or higher”), employment status (“full-time employment/self-employed worker,” “part-time employee,” or “others [student, Unemployed/ after retirement]”), marital status (“married,” “never married,” and “divorced or bereaved”), number of people in the household (including themselves), annual income in 2019, change in current income. The frequency of irregular meals/snacks, skipping breakfast, and eating alone (increased, unchanged, or decreased before April 2020 and after June 2020) was used to assess dietary habit change according to the categories of food expenditure insufficiency.

### Statistical analysis

We mainly presented weighted percentages of variables according to food expense insufficiency status (insufficient food expense for the first time after April 2020, insufficient food expense continued from before April 2020, or not insufficient). As there is a possibility that participants who answered the internet survey were different from the Japanese representative population, the inverse probability weighting (IPW) method using a propensity score (PS) was used to adjust for the bias of the internet survey in the analysis of Group 1 [18]. The propensity score was calculated using a logistic regression model from the pooled dataset of the internet survey and the Comprehensive Survey of Living Conditions of People on Health and Welfare (CSLCPHW) [20], which is regarded as a nationally representative sample of Japanese people. The variables available from CSLCPHW 2016 [20] and JACSIS were used. To calculate PS, area, homeownership, marital status, education, smoking status, and self-rated health were adjusted in the model for participants aged 20–79 years. For participants aged 15–19 years, we adjusted for area, homeownership, education, and self-rated health. The details of this method have been previously described [18]. The variables in Group 2 were not weighted. There were no missing sociodemographic or socioeconomic status variables except annual income and the percentage of their current income compared with their previous income. A total of 5,274 participants in Group 1 and 192 participants in Group 2 answered “unknown” or “not willing to answer” the question about annual income. A total of 6,897 participants in Group 1 and 358 participants in Group 2 did not answer the question on income change. These participants were excluded when the percentage of their annual income was calculated. P-values of each factor were calculated using analysis of variance and the chi-square test among “food expense insufficiency (before April 2020),” “food expense insufficiency (after April 2020, for the first time),” and “no insufficiency.” To examine the multivariable-adjusted odds ratio (AOR) and 95% confidence interval (CI) of “food expense insufficiency for the first time after April 2020” compared to “no insufficiency,” a weighted (or unweighted for Group 2) logistic regression model was constructed. A variable of insufficiency was added to the model as a dependent variable, sociodemographic variables were used as independent variables, and other demographic variables were regarded as confounding factors. As single parents might be at a high risk of food expense insufficiency, we further examined the effect of multiple risk factors in Group 2. We calculated the percentage of single parents with insufficiency, according to the number of risk factors. P-value < 0.05 was considered to be statistically significant and statistical analyses were performed using SAS version 9.4 (SAS Institute Inc., Cary, NC, USA).

## Results

The participant selection process is illustrated in Fig 1. The basic characteristics of the participants are shown in Table 1, and the unweighted results are shown in S1 Table. About half of the participants of Group 1 were men, whereas Group 2 comprised 13.2% men. The percentage of part-time workers was 20.4% in Group 1 and 35.8% in Group 2. The mean ages of Groups 1 and 2 were 48.8 years and 38.5 years, respectively.

**Table 1:** Basic characteristics of participants.

	Group 1* (n = 25,482)	Group 2‡ (n = 1,096)	
Age (mean [SD])	48.8 (17.4)		38.5 (7.7)
Men (%)	49.7		13.2
Area divided by the number of COVID-19 patients until 04/16/2020 (%)			
Over 10 patients/100,000 area	13.9		17.9
5–9.9 patients/100,000 area	34.9		44.6
Less than 5 patients/100,000 area	51.2		37.5
Education level (%)			
Two-year college graduate or lower	64.0		72.6
Bachelor’s degree or higher	35.2		27.5
Job (%)			
Full-time employment/self-employed worker	41.4		57.4
Part-time employment	20.4		35.8
Student	5.6		0.1
Unemployed/ retired	32.5		6.5
Number of people who live with (%)			
1 (participant only)	16.2		0
2	33.7		36.2
3	23.3		29.3
4	17.1		18.7
5	6.6		10.4
≥6	3.3		5.5
Marital status (%)			
Married	63.2		0
Unmarried	23.7		18.0
Divorced or bereaved	13.1		82.0
Annual income in 2019 (%)†			
<1,000,000 yen	5.4		10.2
1,000,000-<4,000,000	32.3		55.1
4,000,000-<7,000,000	32.5		21.6
7,000,000-<10,000,000	17.9		8.3
≥10,000,000	11.9		4.9

SD: standard deviation

* Group 1 was the survey of people living in Japan, and the results from Group 1 were weighted using inverse probability weighting for representativeness; those from Group 2 were not weighted. Fig 1 illustrates the participant selection process.

† Participants who answered “not willing to answer” or “unknown” (n = 5,274 in Group 1 and n = 192 in Group 2), were excluded.

‡179 single parents were from Group 1 and 917 single parents were participants in the single parents’ survey, which was different from the survey for Group 1 but used the same questionnaire. A total of 1096 single parents (179 from Group 1 and 917 from a single-parent survey) were defined as Group 2.

The sociodemographic and socioeconomic status of participants with or without food expense insufficiency during the COVID-19 pandemic in Group 1 and Group 2 is presented in Tables 2 and 3, respectively. The percentages of participants who experienced food expense insufficiency for the first time after April 2020 were 2.9% (weighted) and 5.0% (unweighted) in Groups 1 and 2, respectively. In Group 1, single-parent status, younger age, male sex, low annual income, and living in an area with a higher percentage of COVID-19 cases were statistically significantly associated with experiencing food expense insufficiency for the first time during the pandemic. The AOR for food expense insufficiency was 7.23 (95% CI: 5.40–9.68) for single parents. Regarding employment status, 47% of participants who experienced food expense insufficiency for the first time after April 2020 were part-time employees. Among single parents, age, employment status, and annual income were significantly associated with food expense insufficiency. “The number of people in the household” was a factor that was differently affected in two groups. In Group 1, having five or more people in the household (including the respondents themselves), whereas in Group 2, living with only their child/children (without other adults), especially children under 6 years old, was associated with food expense insufficiency. The income of participants who experienced food expense insufficiency decreased to approximately 60% of their income before the COVID-19 pandemic in both Groups 1 and 2, compared to the approximately 90% of participants who did not experience insufficiency.

**Table 2:** Sociodemographic factors of participants with or without food expense insufficiency during the COVID-19 pandemic in Group 1 (n = 25,482).

	Food expense insufficiency (before April 2020)	Food expense insufficiency (after April 2020, for the first time)	No insufficiency	P-value*	Multivariable AOR (95% Confidence Interval)*§
Number (unweighted)	1014	579	23889		
Number (weighted)	1314	747	23420		
Percentage (weighted)	5.2	2.9	92.0		
Age (mean [SD])	41.2 (19.6)	35.1 (16.2)	49.7 (17.1)	<0.0001	0.93 (0.93–0.94)^|^
Men (%)	51.6	56.4	49.4	0.0004	1.51 (1.26–1.83)
Single parents (yes, %)	10.2	19.5	1.4	<0.0001	7.23 (5.40–9.68)
Area grouped by the number of COVID-19 patients until 04/16/2020 (%)				<0.0001	
Over 10 patients/100,000 area	18.0	24.2	13.3		1.13 (0.87–1.45)
5–9.9 patients/100,000 area	35.8	41.0	34.6		1.47 (1.23–1.77)
Less than 5 patients/100,000 area	46.1	34.8	52.1		Ref
Achieved education level (%)					
Two-year college graduate or lower	69.2	50.3	64.1	<0.0001	0.87 (0.72–1.05)
Bachelor’s degree or higher	29.0	49.4	35.1		Ref
Job (%)					
Full-time employment/self-employed worker	43.3	27.4	41.7	<0.0001	Ref
Part-time employment	22.0	47.1	19.5		4.23 (3.45–5.18)
Student	5.3	6.3	5.6		0.95 (0.64–1.40)
Unemployed/retired	29.4	19.1	33.2		2.67 (2.08–3.44)
Marital status (%)					
Married	48.0	43.2	64.7	<0.0001	Ref
Unmarried	24.6	17.6	23.9		0.37 (0.29–0.47)
Divorced or bereaved	27.4	39.2	11.5		2.10 (1.67–2.64)
Number of people who live with (%)					
Alone	15.4	24.7	15.9	<0.0001	1.16 (0.88–1.53)
2–4	74.5	47.1	74.8		Ref
5-	10.2	28.2	9.3		2.53 (2.06–3.11)
Annual income in 2019 †(mean [SD])	454 (333)	492 (353)	570 (360)		
<1,000,000 yen (%)	9.0	4.8	4.9	<0.0001	7.50 (3.86–14.6)
1,000,000-<4,000,000 (%)	43.6	44.9	32.4		18.9 (11.0–32.7)
4,000,000-<7,000,000 (%)	28.9	20.7	33.4		9.35 (5.30–16.5)
7,000,000-<10,000,000 (%)	16.6	27.3	18.0		14.2 (8.10–24.8)
≥10,000,000 (%)	1.9	2.3	11.3		Ref
Change in current income (compared to before, set before as 100, Mean [MD]) ‡	92.4 (72.3)	62.2 (31.0)	91.0 (22.3)	<0.0001	0.74 (0.72–0.76) ¶

AOR: adjusted odds ratio, SD: standard deviation

* The differences in “food expense insufficiency (before April 2020),” “food expense insufficiency (after April 2020, for the first time),” and “no insufficiency” were compared using analysis of variance or chi-square test.

† Participants who answered “not willing to answer” or “unknown” (n = 5,274) were excluded from the calculation.

‡ The number of missing was 143 in “food expense insufficiency (after April 2020 for the first time),” 327 in “food expense insufficiency (before April 2020),” and 6,427 in “no insufficiency,” all of which were excluded from the analysis of the OR of income change.

§ “food expense insufficiency (after April 2020 for the first time)” was compared to “no insufficiency” in the OR model. Age (5-year increment), sex, education level, job, marital status, annual income category (<1,000,000 yen, 1,000,000-<4,000,000, 4,000,000-<7,000,000, 7,000,000-<10,000,000, ≥10,000,000, ‘not willing to answer” or “unknown”) and areas were adjusted mutually.

|OR for age per 1-year increment

¶ OR of change in current income is per 10 increments and excludes annual income categories from the model.

**Table 3:** Sociodemographic factors of participants with or without food expense insufficiency during COVID-19 pandemic in Group 2 (n = 1,096)*.

	Food expense insufficiency (before April 2020)	Food expense insufficiency (after April 2020 for the first time)	No insufficiency	P-value	Multivariable AOR (95% Confidence Interval) §
Number (N)	86	55	955		
Frequency (unweighted,%)	7.9	5.0	87.1		
Age (Mean [SD])	39.6 (8.8)	35.1 (8.3)	38.6 (7.5)	0.001	0.94 (0.90–0.98) |
Men (%)	18.6	16.4	12.6	0.22	2.08 (0.88–4.92)
Area grouped by the number of COVID-19 patients until 04/16/2020 (%)				0.51	
Over 10 patients/100,000 population area	14.0	10.9	18.6		0.51 (0.20–1.35)
5–9.9 patients/100,000 population area	48.8	49.1	44.0		1.06 (0.58–1.92)
Less than 5 patients/100,000 population area	37.2	40.0	37.4		Ref
Achieved education level (%)					
High school or lower	36.1	45.5	34.8	0.27	1.05 (0.59–1.89)
College or higher	64.0	54.6	65.2		Ref
Job (%)					
Full-time employment/self-employed worker	45.4	30.9	57.7	0.002	Ref
Part-time employment	39.5	49.1	33.8		2.20 (1.14–4.28)
Others (student, unemployed/ retired)	14.1	20.0	8.4		3.80 (1.50–9.60)
Marital status (%)					
Unmarried	18.6	18.2	21.5	0.71	Ref
Divorced or bereaved	81.4	81.8	78.5		2.17 (0.91–5.21)
Live only with child (%)					
With a child/children under 6	12.8	32.7	16.5	0.005	2.38 (1.09–5.18) ||
With a child/children over 6	53.5	40.0	42.3		1.94 (0.89–4.25) ||
With other members	39.7	27.3	41.2		Ref
Annual income in 2019†, Mean (SD)	397 (426)	270 (223)	399 (318)	0.03	
<1,000,000 yen (%)	14.1	20.8	9.2	0.005	3.42 (1.17–9.96)
1,000,000-<4,000,000 (%)	57.8	62.5	54.4		2.55 (1.07–6.09)
≥4,000,000 (%)	40.7	27.3	44.7		Ref
≥7,000,000 (%)	29.1	18.2	28.9		-
Change in current income (compared to before, set before as 100, Mean [SD]) ‡	78.8 (35.7)	61.2 (32.4)	89.5 (26.7)	<0.0001	0.75 (0.68–0.84) ¶

AOR: adjusted odds ratio, SD: standard deviation

* We combined data from participants who answered in the single-parent survey (n = 917) and from participants in Group 1 who met the criteria of a single parent (having two or more people in the household including himself/herself), had no spouse, and had child/children aged 0–14 years old (n = 179). We defined these as Group 2.

† Participants who answered “not willing to answer” or “unknown” (n = 192) were excluded from the calculation.

‡ The number of missing values was 19 in “food expense insufficiency (after April 2020 for the first time),” 28 in “food expense insufficiency (before April 2020),” and 311 in “No insufficiency.” These participants were excluded from the analysis of the OR for income changes.

§ “Food expense insufficiency (for the first time after April 2020)” was compared to “No insufficiency” in the weighted model for OR. Age (5-year increments), sex, education level, job, marital status, annual income category (<1,000,000, 1,000,000–<4,000,000, ≥4,000,000 yen, “not willing to answer” or “unknown”), and areas were mutually adjusted.

| AOR for age is per 1-year increment.

|| The AOR for “Live only with children under and over 6 years old” was 2.14 (1.07–4.29).

¶ OR of change in current income is per 10 increments and excludes annual income categories from the model.

**Fig 2:**
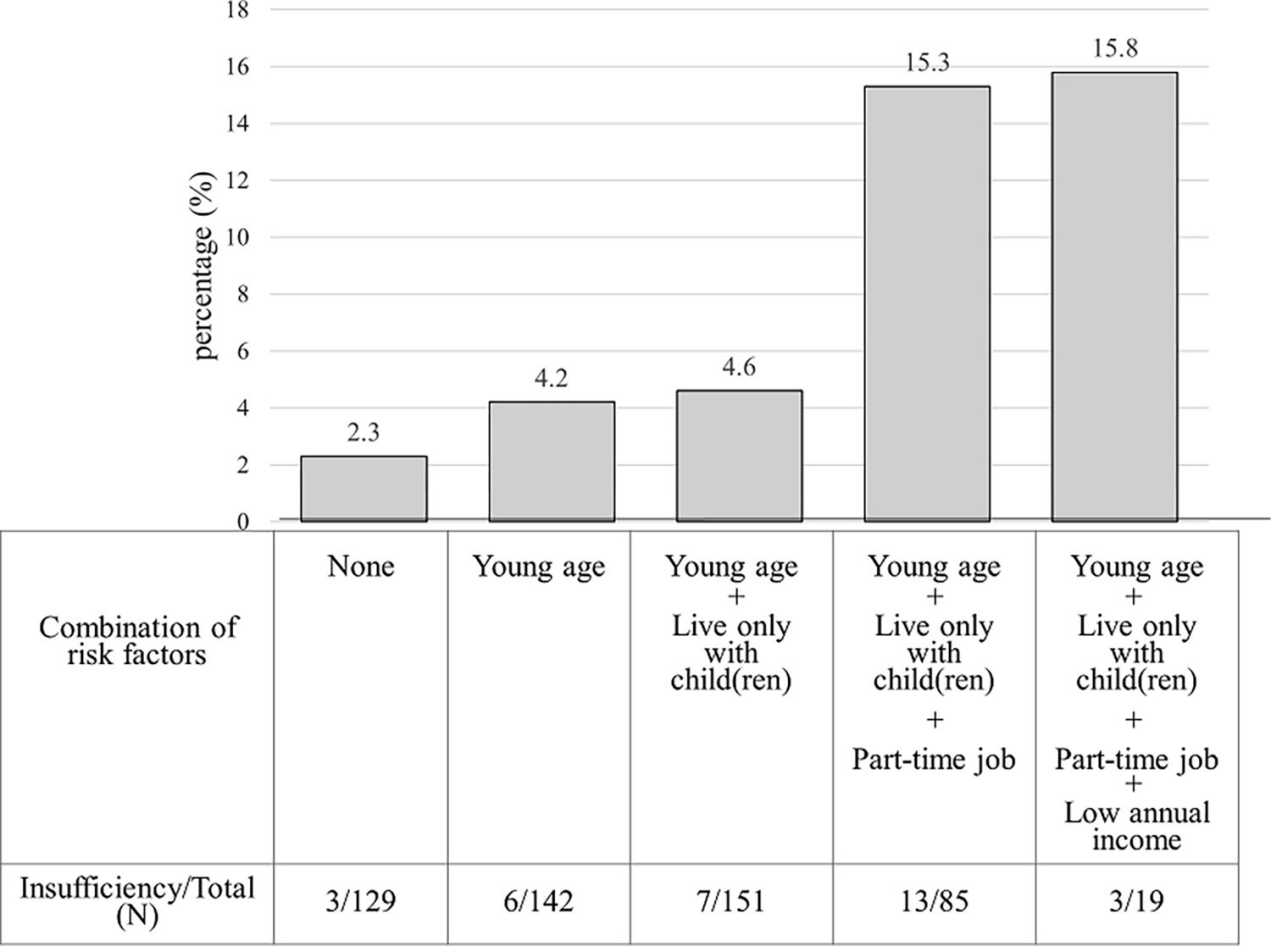
Percentage of single parents with food expense insufficiency after April 2020 for the first time, grouped by the number of associated sociodemographic factors (n = 1096).

Fig 2 shows the percentage of single parents with insufficient food expenses in the group of participants with multiple potential risk factors. These risk factors were identified from among the sociodemographic factors in the results presented in Table 3. Specifically, young age (< 40 years), part-time employment, living with only their child/children, and lower annual income (<1,000,000 yen) were selected. The age cutoff was a round number of the mean age of single parents, whereas the annual income cutoff was determined according to the relative poverty rate (household equivalent income ≤ 1,270,000 yen/year) and our questionnaire categories. Although the number of participants was limited, single parents with multiple risk factors were likely to experience insufficient food expenses. The percentage of participants who experienced insufficient food expenses for the first time after April 2020 was over 15% among single parents with three or four risk factors, compared to approximately 2% among those with no risk factors. The percentage of participants who did not change their dietary habits during the “stay-at-home” period (April and May 2020) was low in the group of participants who experienced insufficiency for the first time, compared to the group that did not experience insufficiency (S2 Table).

## Discussion

This study explored the sociodemographic characteristics of participants who experienced food expense insufficiency during the COVID-19 pandemic. The decrease in income was larger in the food expense insufficiency group than in participants who did not experience insufficiency. Being a “single parent” is one of the strongest risk factors for food expense insufficiency. Sociodemographic factors such as young age, part-time employment, having five or more members in the household in Group 1, and living with only their child or children (especially children under six years old) in Group 2 were likely to increase the vulnerability of a group and pose a high risk of facing food expense insufficiency in an emergency like the COVID-19 pandemic. As food insecurity has rarely been focused on in epidemiological surveys in Japan so far, this study’s findings on food expense insufficiency will be crucial for the public health sector to detect and prevent severe food insecurity, especially for high-risk single-parent families.

Although our survey was conducted online, our results are likely to reflect the actual situation in Japan in terms of capturing a small annual income group. The relative poverty rate was 10.1% in the National Survey of Family Income and Expenditure in 2009 and 15.4% in the Comprehensive Survey of Living Conditions in 2018 in Japan [16, 21]. The half of the disposable income was regarded as the cutoff value for poverty in the Comprehensive Survey of Living Conditions in 2018, which was 1,270,000 yen/year. In our results, the percentage of people who had an annual income lower than 2,000,000 yen in 2019 was 11.5% (weighted) in Group 1 and 24.2% (unweighted) in Group 2; these values are close to those of the abovementioned national surveys.

The results of this study are consistent with those of previous studies and surveys. According to the Comprehensive Survey of Living Conditions in 2018 in Japan, the relative poverty rate among households with single adults and child/children was 48.1%, which was higher than that of households with more than two adults and child/children (12.4%) [16]. This is consistent with our result that single parents were vulnerable to food expense insufficiency; among single parents, those with low annual income and those living in a household with a single adult and child/children were found to be at high risk of being in a state of food expense insufficiency. Previous studies have shown that being a single parent or having a low income is associated with food insecurity or insufficiency [22, 23]. These previous studies were performed mainly in countries where the government had conducted the food insecurity survey; our paper, on the other hand, is worth reporting because there is scarce evidence regarding a dimension of food insecurity in Japan due to the lack of official surveys for food insecurity. Globally, the impact of COVID-19, even in countries with very low poverty rates such as Japan, suggests that measures should be taken in other countries. Moreover, being a part-time employee alone is known to be a risk factor for insufficiency [24], but our nuanced analyses showed that the combination of part-time employment and living status (single parents living only with their children) enhanced this risk. Therefore, individuals with multiple risk factors should receive more attention and support.

As food insecurity has various aspects [15], ideally, it should be tested through the use of several questions, such as the ten questions used by the USDA to assess household food insecurity [25, 26]. The USDA’s measurement of food insecurity features economic and social factors, and the questions cover various dimensions of food insecurity, including feelings of uncertainty, households not having enough food to eat, not being able to meet the perceived needs for a balanced diet, and experiences of hunger and weight loss. Although this study only examined the lack of food expenses and did not explore all the dimensions of food insecurity, food expense is known to be correlated with the food insecurity score [27]. Therefore, although further research using a more comprehensive questionnaire on food insecurity is required, this study reveals some important aspects of the status of food insecurity in Japan during the COVID-19 pandemic.

One of the strengths of this study was its timing: It was conducted during the COVID-19 pandemic, thus reducing the chances of recall bias. Moreover, our Internet survey was able to reach participants who could not physically participate in the survey. However, internet surveys also have limitations. Although we used the weighted model to correct for some bias, people who could not use the internet were not included in this survey, which may have caused selection bias. Another limitation was that food insecurity in this survey was explored through only one question regarding the food expense insufficiency. As the self-reported food expense insufficiency in this questionnaire was subjective and not validated, a difference in “insufficiency” among individuals may exist. One question alone is insufficient to capture the different aspects of food insecurity, and self-reported annual income may contain reporting errors. Further research using a comprehensive food insecurity questionnaire is required. Furthermore, it is unknown whether this shortage of food expenditure lasts long term or affects nutritional consumption in Japan. As this study was cross-sectional and considered the status at only one point, the participants’ status may have changed. Although further research is required in these respects, this study revealed some aspects of food insecurity in a high-income country that will be useful for public health and nutrition policymaking.

In conclusion, approximately 3% of participants experienced insufficient food expenses during the COVID-19 pandemic for the first time, and factors such as being a single parent, young age, part-time employment, and low income were significantly associated with food expense insufficiency. Single parents who do not live with other adults are at risk of facing insufficient food expenses, and those who exhibit multiple risk factors are even more vulnerable. Both public health safety nets, such as food supply for these people, and a national survey on food insecurity are urgent, even in a high-income country like Japan.

## Supporting information

S1 Table Results of sociodemographic factors of participants with or without food expense insufficiency in an unweighted model* (n = 25,482). (DOCX)

S2 Table Change in dietary habit during “stay-home” period (April and May 2020) in Japan according to the category of food expense insufficiency (n = 25,482). (DOCX)
