# Supplementary material for: Socioeconomic and sociodemographic factors associated with food expense insufficiency during the COVID-19 pandemic in Japan
Source: PLoS One. 2022 Dec 15;17(12):e0279266. doi: 10.1371/journal.pone.0279266 (PMC9754285; doi:10.1371/journal.pone.0279266)
Supplement: S1 Table — (DOCX) [file pone.0279266.s001.docx]

**S1 Table** Results of sociodemographic factors of participants with or without insufficiency of food expense in an unweighted model* (n=25,482)

|  | Insufficiency of food expense (before April 2020) | Insufficiency of food expense (after April 2020 for the first time) | Not insufficient | P-value | Multivariable adjusted OR |
| --- | --- | --- | --- | --- | --- |
| Number (unweighted) | 1014 | 579 | 23889 |  |  |
| Percentage (unweighted) | 4.0 | 2.3 | 93.8 |  |  |
| Age (Mean (SD)) | 42.2 (16.0) | 39.5 (16.3) | 49.3 (17.3) | <0.0001 | 0.80 (0.77-0.83) |
| Men (%) | 46.3 | 38.9 | 50.1 | <0.0001 | 0.75 (0.62-0.90) |
| Area grouped by the number of Covid-19 patients until 2020/04/16 (%) |  |  |  | 0.51 |  |
| Over 10 patients/100,000 area | 19.3 | 20.6 | 20.1 |  | 1.05 (0.83-1.33) |
| 5-9.9 patients/100,000 area | 43.6 | 46.1 | 45.4 |  | 1.07 (0.89-1.30) |
| Less than 5 patients/100,000 area | 37.1 | 33.3 | 34.5 |  | Ref |
| Achieved education level (%) |  |  |  |  |  |
| Two-year college graduate or lower | 62.2 | 56.1 | 51.5 | <0.0001 | 1.22 (1.02-1.45) |
| Bachelor’s degree or higher | 37.0 | 43.2 | 48.3 |  | Ref |
| Job (%) |  |  |  |  |  |
| Full-time employment/self-employed worker | 36.2 | 39.4 | 43.4 | <0.0001 | Ref |
| Part-time employment | 25.5 | 20.4 | 17.2 |  | 1.20 (0.94-1.54) |
| Student | 10.0 | 14.0 | 6.6 |  | 1.16 (0.85-1.59) |
| Unemployed/ after retirement | 28.3 | 26.3 | 32.8 |  | 1.14 (0.89-1.45) |
| Marital status (%) |  |  |  |  |  |
| Married | 47.3 | 52.7 | 60.5 | <0.0001 | Ref |
| Unmarried | 40.2 | 37.5 | 30.1 |  | 0.53 (0.42-0.67) |
| Divorced or bereaved | 12.4 | 9.8 | 9.5 |  | 1.13 (0.83-1.54) |
| Number of people who live with (%) |  |  |  |  |  |
| alone | 26.1 | 22.8 | 19.3 | <0.0001 | 1.21 (0.93-1.56) |
| 2-4 | 63.1 | 65.1 | 73.4 |  | Ref |
| 5- | 10.8 | 12.1 | 7.4 |  | 2.68 (2.19-3.28) |
| Annual income in 2019 †(mean (SD)) | 454 (333) | 492 (353) | 570 (360) |  |  |
| <1,000,000 yen (%) | 14.2 | 12.1 | 4.8 | <0.0001 | 3.58 (2.57-5.00) |
| 1,000,000-<4,000,000 (%) | 43.2 | 37.0 | 31.7 |  | 2.26 (1.78-2.88) |
| 4,000,000-<7,000,000 (%) | 27.3 | 31.7 | 32.8 |  | 1.51 (1.18-1.93) |
| 7,000,000-<10,000,000 (%) | 10.5 | 12.9 | 18.3 |  | 1.08 (0.79-1.48) |
| >=10,000,000 (%) | 4.8 | 6.3 | 12.4 |  | Ref |
| Change in current income (compared to before, set before as 100, Mean (SD)) | 74.8 (33.1) | 65.1 (33.1) | 91.3 (22.9) | <0.0001 | 0.76 (0.74-0.78) |
| Having loan/mortgage (yes, %) | 55.7 | 56.5 | 31.9 | <0.0001 | 2.63 (2.19-3.16) |

* All the analysis were same as Table 2 except using inverse probability weighting method.

**S2 Table** Change in Dietary habits during “stay-home” period (April and May 2020) in Japan according to the category of food expense insufficiency (n=25,482)

|  | Insufficiency of food expense (after April 2020 for the first time) | Insufficiency of food expense (before April 2020) | Not insufficient | p-value |
| --- | --- | --- | --- | --- |
| Number (unweighted) | 579 | 1014 | 23889 |  |
| Number (weighted) | 747 | 1314 | 23420 |  |
| Frequency of having irregular meals/snacks |  |  |  | <0.0001 |
| Increase | 42.0 | 25.0 | 14.0 |  |
| Unchanged | 49.0 | 66.5 | 78.1 |  |
| Decrease | 9.0 | 8.5 | 7.9 |  |
| Frequency of skipping breakfast |  |  |  | <0.0001 |
| Increase | 30.2 | 26.3 | 3.3 |  |
| Unchanged | 58.8 | 68.1 | 89.9 |  |
| Decrease | 11.1 | 5.6 | 6.9 |  |
| Frequency of eating alone |  |  |  | <0.0001 |
| Increase | 21.3 | 11.3 | 11.4 |  |
| Unchanged | 69.3 | 80.6 | 82.2 |  |
| Decrease | 9.5 | 8.2 | 6.4 |  |
| Cost for eating out or taking out (yen) |  |  |  |  |
| Before Jan 2020, Mean (SD) | 6990 (12232) | 6284 (9571) | 7029 (13372) | <0.0001 |
| Before Jan 2020, Median | 3000 | 5000 | 3000 |  |
| Aug 2020, Mean (SD) | 4716 (9086) | 5309 (8588) | 5195 (12075) | 0.008 |
| Aug 2020, Median | 3000 | 3000 | 2000 |  |
| Change | 2274 (10421) | 974 (6886) | 1834 (11178) | <0.0001 |

Values in the table are shown in percentage except cost for eating out.
