# Supplementary material for: Socioeconomic and sociodemographic factors associated with food expense insufficiency during the COVID-19 pandemic in Japan
Source: PLoS One. 2022 Dec 15;17(12):e0279266. doi: 10.1371/journal.pone.0279266 (PMC9754285; doi:10.1371/journal.pone.0279266)
Supplement: S2 Table — (DOCX) [file pone.0279266.s002.docx]

**S2 Table** Change in Dietary habits during “stay-home” period (April and May 2020) in Japan according to the category of food expense insufficiency (n=25,482)

|  | Insufficiency of food expense (after April 2020 for the first time) | Insufficiency of food expense (before April 2020) | Not insufficient | p-value |
| --- | --- | --- | --- | --- |
| Number (unweighted) | 579 | 1014 | 23889 |  |
| Number (weighted) | 747 | 1314 | 23420 |  |
| Frequency of having irregular meals/snacks |  |  |  | <0.0001 |
| Increase | 42.0 | 25.0 | 14.0 |  |
| Unchanged | 49.0 | 66.5 | 78.1 |  |
| Decrease | 9.0 | 8.5 | 7.9 |  |
| Frequency of skipping breakfast |  |  |  | <0.0001 |
| Increase | 30.2 | 26.3 | 3.3 |  |
| Unchanged | 58.8 | 68.1 | 89.9 |  |
| Decrease | 11.1 | 5.6 | 6.9 |  |
| Frequency of eating alone |  |  |  | <0.0001 |
| Increase | 21.3 | 11.3 | 11.4 |  |
| Unchanged | 69.3 | 80.6 | 82.2 |  |
| Decrease | 9.5 | 8.2 | 6.4 |  |
| Cost for eating out or taking out (yen) |  |  |  |  |
| Before Jan 2020, Mean (SD) | 6990 (12232) | 6284 (9571) | 7029 (13372) | <0.0001 |
| Before Jan 2020, Median | 3000 | 5000 | 3000 |  |
| Aug 2020, Mean (SD) | 4716 (9086) | 5309 (8588) | 5195 (12075) | 0.008 |
| Aug 2020, Median | 3000 | 3000 | 2000 |  |
| Change | 2274 (10421) | 974 (6886) | 1834 (11178) | <0.0001 |

Values in the table are shown in percentage except cost for eating out.
